# A Bad Memory

**Published:** 1856-01

**Authors:** 


### A BAD MEMORY

Is a man's own fault; it is simply a want of attention; it is the result of a "Don't care" disposition.

1. Think of a thing distinctly within five minutes after you have first made an impress of it on the mind, and you will seldom fail of its recollection.

2. The *memory*, like a true friend, is made the firmer by being trusted; noting down trifling things, is the very way to destroy what remnant of memory you have.

3. Method improves and assists the memory greatly.

We will illustrate, by a practice of our own, a long time ago. For the purpose of preventing the discontinuance of our papers by failure to remit the subscription price, as well as avoiding the "advance" on tardy subscribers, we made all our subscriptions *due* on New Year's Day, and remitted the same during the Christmas holidays, *for the coming year*. This is simply a practical suggestion about one of the little things of life; attention to it would save hundreds of thousands of dollars every year to Editors as well as to subscribers; would save a loss of time, and feeling, and patience, in dunning and being dunned, not readily estimated.

